# Gas Exchange, Water Use Efficiency, and Biomass Partitioning among Geographic Sources of *Acer saccharum* Subsp. *saccharum* and Subsp. *nigrum* Seedlings in Response to Water Stress

**DOI:** 10.3390/plants10040742

**Published:** 2021-04-10

**Authors:** Richard J. Hauer, Hongxu Wei, Andrew K. Koeser, Jeffrey O. Dawson

**Affiliations:** 1 College of Natural Resources, University of Wisconsin-Stevens Point, Stevens Point, WI 54481, USA; 2 Northeast Institute of Geography and Agroecology, Chinese Academy of Sciences, Changchun 130102, China; weihongxu@iga.ac.cn; 3 Gulf Coast Research and Education Center, University of Florida, 14625 CR 672, Wimauma, FL 33598, USA; akoeser@ufl.edu; 4 Department of Natural Resources and Environmental Sciences, University of Illinois at Urbana/Champaign, Urbana, IL 61801, USA; jdawson2@illinois.edu

**Keywords:** abiotic stress, forestry, plant selection, tree physiology, urban forestry

## Abstract

Responses to water stress were measured for sugar maple (*Acer saccharum* subsp. *saccharum* Marshall) sources from Oklahoma (Caddo sugar maple), Missouri, Tennessee, Ontario, and a black maple (*Acer saccharum* subsp. *nigrum* F. Michx.) source from Iowa. Seedling sources were selected for differences in temperature and precipitation of their geographic origins. Seedlings were preconditioned through moist (watered daily) or dry (watered every 4–7 days) cycles and then exposed to prolonged water stress. As water stress increased, dry preconditioned 17-week-old sugar maple seedlings from Oklahoma, Missouri, and Tennessee, sources from warmer, and/or drier climates with greater restrained photosynthesis, stomatal conductance, and water use efficiency than those from cooler and moister climates. Under imposed water stress, the Ontario and Iowa sourced seedlings increased their root to shoot ratios and decreased their specific leaf area, mechanisms for drought avoidance. However, no corresponding changes in these values occurred for Oklahoma, Missouri, and Tennessee sources and for the variable of leaf wilting across all sources. Results from this study suggest greater tolerance of water stress in the Oklahoma, Missouri, and Tennessee ecotypes from the western and southern range of sugar maple resulted primarily with water use efficiency (WUE) rather than other water stress coping mechanisms. Findings from this study provide evidence to support selection of sugar maples sources for forestation.

## 1. Introduction

Sugar maple (*Acer saccharum* Marshall) trees provide wildlife benefits; serve as tribal, provincial, state, and national cultural symbols; are a source for maple syrup; and are harvested for valuable wood products [1]. Additionally, sugar maple is commonly planted as urban and residential trees for their shade, stature, beautiful fall leaf color, and pleasing growth habit [2]. Sugar maple trees (*Acer saccharum* Marshall subsp. *saccharum*) have been regarded as susceptible to long-term water stress [3,4,5]. This is manifested through drought in forests in North America [6,7,8,9]. Water stress leading to maple decline and death has also been reported in urban and peri-urban landscapes [10,11,12,13].

Sugar maple trees decline as a result of water stress has commonly been observed in native and planted stands, and ecotypes from the southwestern part of the species’ range have been observed to possess greater resistance to water deficits than sugar maple ecotypes from the northern and eastern parts of the species’ range [14,15,16]. The Caddo sugar maple (*Acer saccharum* “Caddo”) from central Oklahoma USA is an example of a sugar maple ecotype with potentially greater drought resistance [17,18,19,20]. Thus, we selected and tested sugar maple seedlings representing presumptive ecotypes sourced from locales that varied in temperature and precipitation, and relative evapotranspiration potential [21].

Black maple (*A. saccharum* subsp. *nigrum*) trees, particularly from Iowa, USA, have been speculated to possess greater resistance to water deficits compared to associated sugar maple trees and sugar maple from parts of its eastern range [22,23,24]. Such suggestions are based on topographic features and the fact that the average climatic conditions within black maples’ range are warmer and drier than much of sugar maples’ total range [25,26,27]. Several woody plant reference books focusing on tree selections for cultivation have suggested that black maple has greater tolerance to water stress than sugar maple [28,29]. In contrast, Dirr [2] observed that sugar and black maple trees in urban landscapes do not distinguishably differ in response to water deficits. Niinemets and Valladares [30] reviewed drought tolerance reports on 806 woody plants species from the Northern Hemisphere, ranking (1 to 5 scale, 5 most tolerant) black maple more tolerant (3.35 ± 0.35 SE) than sugar maple (2.25 ± 0.25 SE). Conflicting reports indicate a need for additional ecophysiological studies to elucidate functional and structural traits that might explain inconsistent observational reports of ecotypic variation in tolerance to water stress of sugar and black maples [31]. Further, Skepner and Krane [32] identified through RAPD-PCR analysis that black and sugar maple while genetically should be classified as subspecies, a geographical source distinction was detected resulting from a putative local environment effect.

Black and sugar maple trees in general grow naturally on a variety of sites, however, both prefer well-drained, mesic soils [1,33]. When they naturally occur in the same locale, black maple trees tend to occur on the more mesic sites, whereas sugar maple concurrently occurs on relatively drier sites [24,34,35]. For example, in western Indiana, USA, and eastern Illinois, USA, black maple trees are typically restricted to more mesic deeper soils than sugar maple trees [36]. Furthermore, black maple trees in Iowa typically occur on north facing slopes or in areas with ample soil moisture, rather than on more xeric sites [37]. Thus, published reports differ in their assessments of sugar and black maples’ general and specific abilities to withstand drought stress.

Field and greenhouse studies have been used to investigate water stress tolerance of sugar and black maples from different seed sources. Sources from the southwestern portion of the range of the species tend to have greater potential evapotranspiration than those from the northeastern part of the range [21]. Sugar maple trees from the southwestern part of its range were reported to have greater survival, resistance to water stress, and less leaf scorching than sources from the species eastern range [14,16,18]. Pair [18] observed less negative predawn water potential, less leaf scorch, and less leaf tatter following a summer drought in the Caddo sugar maple (Oklahoma source) and sugar maple cultivars (“Commemoration” and “Legacy”) sourced from the species’ southern range, than sugar maple cultivars (“Bonfire” and “Green Mountain”) from more eastern locations in North America. An Iowa black maple source (“Green Column”) was intermediate. Graves [23] determined in a seedling study that a west central Iowa black maple source had a greater capacity to withstand water stress than a native Minnesota, USA, sugar maple source from 275 km to the north and a location with relatively cooler summer temperatures (~1.5 °C).

In additional to any potential genetic differences, questions remain as to whether mild water stress more common to certain sites can precondition changes in sugar and black maple to water stress [38,39]. Preconditioning can lead to increased physiological activity during water stress, changes in biomass portioning, and morphological development [40]. However, there are no reports in the literature on the effects of water stress preconditioning on key sugar maple seedling physiological and morphological adaptations in the context of ecotypic variation. 

The aim of this study was to compare and determine seedling physiological and biomass allocation responses of sugar maple and black maple sources to water stress with or without water-stress preconditioning treatments. The following questions were posed: (1) how do sugar maple ecotypes vary in biomass partitioning; (2) what are the photosynthetic rates of sugar maple seedlings under contrasting soil moisture regimes; (3) what are the other foliar gas exchange rates of sugar maple seedlings under contrasting soil moisture regimes; (4) do sugar maple ecotypes differ in seedling water use efficiency (WUE) in response to water stress; and (5) do maple seed geographic sources produce seedlings that differ in water stress response to mild water stress preconditioning? We hypothesized that the southern and western sugar maple seedlings sourced from warmer, often drier locales in Oklahoma (Caddo sugar maple), Missouri, and Tennessee possess greater water stress resistance than sources from Ontario and Iowa (black maple). The Caddo sugar maple was hypothesized to have the highest net photosynthetic rate and water use efficiency (WUE) with increasing exposure to increasing water stress. Water use efficiency and net photosynthesis were hypothesized to decline more rapidly with water deficits in sugar maple seedlings from Ontario. Black maple was hypothesized to be more sensitive to water stress than the sugar maple sources from Missouri, Tennessee, and Oklahoma. We hypothesized that natural selection for water stress tolerance would have been most pronounced in maples from locations having drier, warmer climates.

## 2. Materials and Methods

### 2.1. Seed Source, Germination, and Establishment

Experimental plants were grown from seed obtained from Sunshine Nursery (Clinton, OK) for the Oklahoma (Caddo) source originating from Red Rock Canyon; from Sheffield’s Seed Company (Locke, NY, USA) for the Tennessee, Missouri, and Ontario sources; and from Smith Nursery Co. (Charles City, IA, USA) for the Iowa black maple source (Table 1). The seedlings exhibited morphological traits consistent with their taxonomic assignments and presumptive ecotypes. Stipules were observed on black maple at the base of the petiole, leaves had three prominent lobes and drooped, and the leaf underside was pubescent. The Ontario source’s seedling leaves were generally five lobed with narrow sinuses, glabrous underneath, and were the thinnest of all geographic sources. The Missouri source had 3–5 lobed leaves with intermediate pubescence. Leaves from the Tennessee source had the most pubescence producing a whitish appearance along the mid rib and veins, and were 3–5 lobed, with deep sinuses. The Caddo source’s leaves were generally 3–5 lobed, had a thicker waxy appearance, and an intermediate level of pubescence.

**Figure 1:**
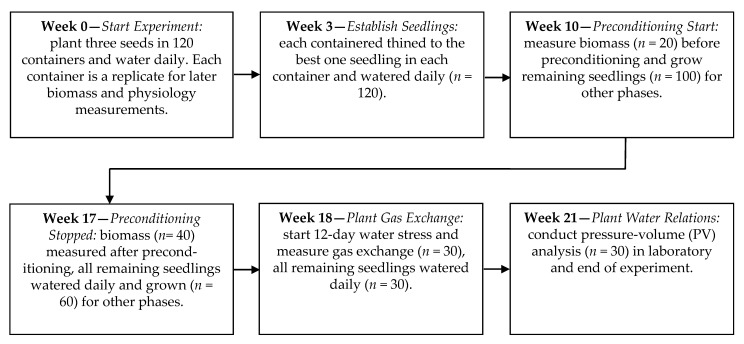
Timeline of experiment with key phases (in italics, e.g., *Start Experiment*) and outcomes. Preconditioning was the daily watering or watering of seedlings after exhibiting leaf wilting, and then re-water and continue this during the 7-week preconditioning period. Each biomass measurement (week 10 and week 17) was a destructive harvest for four replications per each of the five seed sources and preconditioning treatments. Gas exchange and plant water relations—each had three replications per each seed source and preconditioning combination. Each replication was in one container.

In late October, seeds were surface sterilized in 10% H_2_O_2_ for 15 min and soaked in dH_2_O for 14 days at 3–4 °C. Moist seeds were stratified in the dark at 3–4 °C for 45 days [41]. Three seeds per container were planted in late December in 2.4 L (10 cm × 30 cm PVC plastic pipe with 2-mm nylon mesh over open bottoms to allow drainage). These plant containers were filled with a steam pasteurized 2:2:1 (*v/v/v*) peat moss, coarse sand, and Drummer silty clay loam soil mix [42]. Seedlings were thinned from pots after 3 weeks leaving 1 per container. All seedlings were grown for an additional 7 weeks and watered daily or as needed to maintain moist soil prior to allocation to experimental subsets. A total 120 containers were grown in a random arrangement of a greenhouse bench. Experimental plants were randomly allocated to subsets as described later for destructive biomass and morphology measurements before (*n* = 20) and after moisture stress preconditioning (*n* = 40), plant gas exchange measurements (*n* = 30), and plant water relation (*n* = 30) measurements (Figure 1).

Supplemental light from Sylvania L41000 metal halide lamps (GTE Sylvania, Inc., Manchester, NH, USA) was provided automatically when ambient radiation decreased below 400 µmol × m^−2^ s^−1^ of photosynthetic photon flux density (PPFD) as measured with a Li-Cor Quantum Sensor (Li-Cor, Lincoln, NE, USA) during a 16-h photoperiod. A 16-h photoperiod was used to promote active growth and minimize bud set in sugar maple [43,44]. Greenhouse temperatures were 22 °C (days) and 19 °C (nights) ±3 °C. During the establishment period (10 weeks), seedlings were watered daily or as needed and fertilized weekly with a 20:20:20 (nitrogen (N):phosphorus (P):potassium (K)) Peter’s brand (Allentown, PA) fertilizer solution (N at 473 ppm) supplemented with a full-strength Hoagland’s [45] micronutrient solution using Sprint 330 (BASF, Research Triangle Park, NC, USA) as an iron chelating agent.

### 2.2. Seedling Preconditioning

Ten-week-old seedlings grown as described above were subjected to two preconditioning treatments in early March for 7 weeks. Only actively growing seedlings that had not set a terminal bud were selected for random allocation to treatments for all five seedling sources. Seedlings were preconditioned through either a moist regime consisting of daily watering to soil capacity or subjected to a series of drying cycles for the dry regime in which all seedlings were then re-watered to soil water-holding capacity for 2 days after flaccid leaves were observed. Dry cycles (watering withheld) were used to mimic natural water stress and to see if it could induce greater tolerance to water stress. Dry cycles lasted between 4 and 5 days. Seedlings were fertilized three times during the preconditioning period with the fertilizer solution described above.

### 2.3. Seedling Mass and Leaf Area

The dry mass of leaf, stem, shoot (leaf and stem combined), and root tissue along with leaf area were measured for a subset sample of four actively growing seedlings per treatment prior to (*n* = 20) and after preconditioning (*n* = 40). Seedling mass was determined from tissue dried (for 48–72 h) to consistent mass at 70 °C. Total leaf area per seedling was estimated using a leaf area meter (Li-Cor Model 3100, Lincoln, NE, USA). Seedling height was measured after preconditioning.

### 2.4. Plant Water Relations and Gas Exchange

Following the 7-week preconditioning period, seedlings in all 10 treatment combinations (2 preconditioning regimes × 5 seed sources) were watered daily for 7 days. Watering was then stopped and then daily gas exchange measurements including net photosynthesis (A, µmol CO_2_ m^−2^ s^−1^), stomatal conductance (G_s_, mol H_2_O m^−2^ s^−1^), transpiration (E, mol H_2_O m^−2^ s^−1^), intercellular CO_2_ concentration (Ci, ppm), ambient CO_2_ concentration (Ca, ppm), and instantaneous water use efficiency (WUE, µmol mol^−1^) derived from A E^−1^ occurred on 3 seedlings per treatment using a Li-Cor model 6200 portable photosynthesis system, and model 6250 infrared gas analyzer (LI-Cor Inc., Lincoln, NE, USA) for 3 replications (*n* = 30) for these measurements. Measurements were made on the first fully expanded leaf, corresponding to a plastochron age of 4–6. These leaves had formed during the preconditioning period. Measurements were made daily between 9:30 and 10:30 a.m. central standard time in an environmentally controlled research greenhouse located in Urbana, Illinois (40.1106 N, 88.2073 W). This time period corresponded to peak CO_2_ exchange rates during trials with seedlings tested under both moist and dry conditions (data not shown). Measurements were stopped after day 12 as net photosynthesis closely approached 0 for all source and treatment combinations. Diurnal measurements also occurred on days 0, 3, 5, 6, 7, and 9 with all plants measured every 2 h during daylight hours. Artificial lighting was employed on days 1 and 6 to compensate for cloudy conditions to maintain a light intensity at seedling level at a minimum PPFD of 400 µmol m^−2^ s^−1^. This was a level found from previous testing to result in maximum photosynthetic rate (data not shown). Gas exchange measurements were derived from the mean of three samples each over a 10-s period from a leaf in a quarter-liter chamber for approximately 1 min.

Daily measurements of leaf water potential were not taken due to phloem bubbling which obscures end-point recognition of xylem exudation of the petiole in sugar maple and also not to reduce leaf area of seedlings during this measurement period [5], necessitating stem rather than petiole measurements of water potential. Stem samples with leaves were used for pressure-volume (PV) analysis to estimate leaf water potential and the osmotic potential at full turgor (Y_100_), osmotic potential at zero turgor (Y_0_), and relative water content at zero turgor. Seedlings were watered daily for 4 weeks after preconditioning was terminated. Seedlings (*n* = 30) used for PV analysis were watered to soil capacity and placed in a dark room overnight. The following morning, 10 cm long stems that contained 6–8 leaves were cut under low light conditions, weighed immediately afterwards to estimate the saturated tissue mass, an estimate of the water potential at full saturation was made, and the free transpiration PV technique was used to estimate PV parameters [46] using a pressure chamber (Model 3005, Soil Moisture Equipment Corp., Santa Barbara, CA, USA).

### 2.5. Experimental Design and Statistical Analysis

The experimental design was a 5 × 2 factorial combination that included seedlings from five seed sources that were preconditioned through either a moist or dry watering regime. Containers were arranged in a completely randomized manner on greenhouse benches. All statistical analyses were performed using IBM SPSS version 25 (IBM Corp., Armonk, NY, USA). Mean differences in biomass portioning and leaf water relations were analyzed using an ANOVA. A generalized least squares repeated measures mixed model was used to analyze temporal changes in gas exchange measurements. Mean differences during each day for leaf water relations and gas exchange were analyzed using ANOVA. Tests for model assumptions were made and no correction of data was needed. Means were separated in all cases with a Fisher’s Protected LSD at the a = 0.05 probability level for biomass measurements. A Duncan test was used for the leaf water relations measurements due to an unequal sample size.

## 3. Results

### 3.1. Seedling Mass and Height

Prior to preconditioning, 10-week-old seedlings were similar in total leaf area, leaf mass, stem mass, shoot mass, and root to shoot (R:S) ratio (Table 2). Root mass was significantly greater (*p* = 0.047) in the Iowa (black maple) source, nearly two times more than the other four ecotypes. Specific leaf area was greatest (*p* = 0.029) in Caddo and Tennessee sources.

Significant differences occurred among sources in shoot and root growth of 17-week-old seedlings after preconditioning (Table 3). The exception was no difference in plant height among sources (*p* = 0.174). Seed source had the greatest significance (*p* < 0.02) associated with the difference in the mass of leaf, stem, shoot, and root attributes. Preconditioning had a significant effect (*p* < 0.05) on leaf area and leaf mass attributes with a 42% decline in leaf area and a 26% decrease in leaf mass of black maple. Likewise, black maple had less stem and shoot mass. Root mass was 2.3 times greater (*p* = 0.002) in black maple than the sugar maple sources. The R:S of the Iowa black maple and Ontario sugar maple sources prior to preconditioning (10 weeks) and under moist preconditioning (17 weeks) were similar (Table 2 and Table 3). The Iowa and Ontario sources increased (*p* = 0.002) their R:S and decreased (*p* < 0.001) their specific leaf area (SLA) under the dry preconditioning regime. While the Caddo, Missouri, and Tennessee sources R:S and SLA did not statistically change, resulting in a significant (*p* = 0.002) source by preconditioning interaction. Black maple apparently reduced shoot mass and proportionally allocated biomass to root growth in response to preconditioning water stress. There was no discernible difference among maple seedlings from all sources in leaf wilting observed among sources during a dry cycle.

### 3.2. Plant Water Relations and Gas Exchange

#### 3.2.1. Daily Gas Exchange Analysis

**Figure 2:**
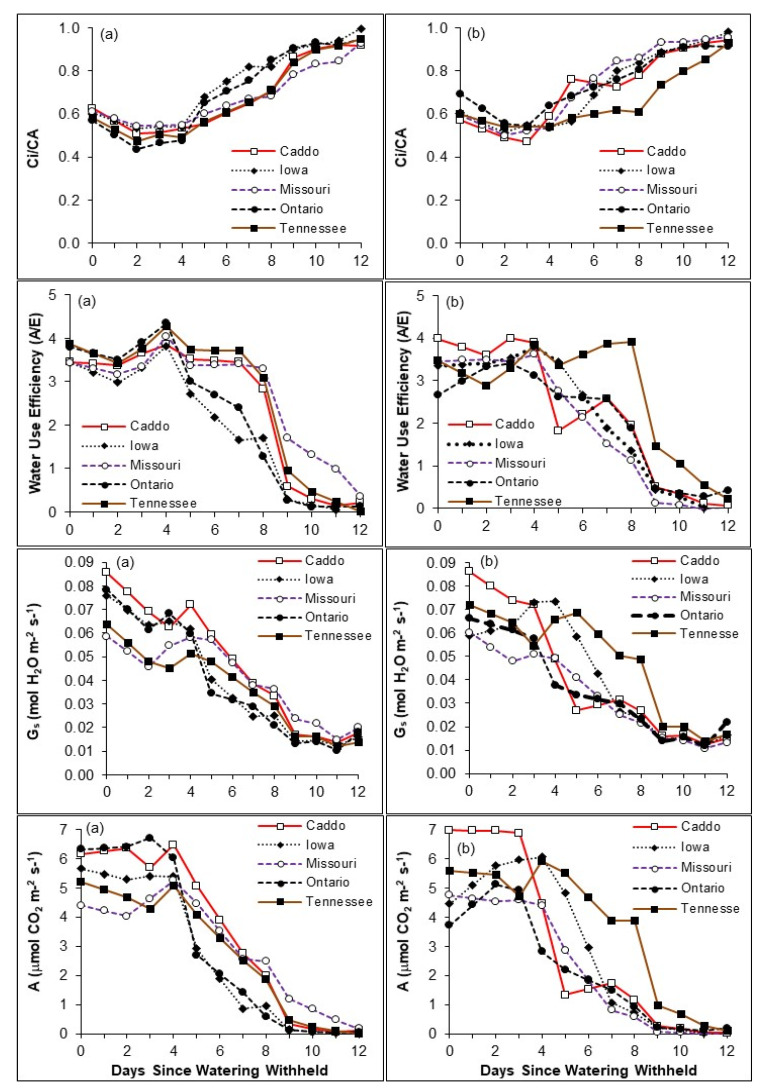
Daily changes in net CO_2_ assimilation (A), stomatal conductance to (G_s_), instantaneous water use efficiency (WUE), and intercellular to ambient CO_2_ concentrations (Ci/Ca) in 10-week-old *Acer saccharum* subsp. *saccharum* and *Acer saccharum* subsp. *nigrum* (Iowa) seedlings, preconditioned during the last 7-weeks under (**a**) dry or (**b**) moist regime and then subjected to no watering over 12 days.

The repeated measures analysis showed all seed sources exhibited significant (*p* < 0.001) declines in A, G_s_, E, and WUE and an increase in Ci and Ci/Ca over time as water was withheld (Figure 2). A significant day × source interaction occurred in A (*p* = 0.002), G_s_ (*p* < 0.001), and E (*p* < 0.001). A significant (*p* < 0.05) day × source × preconditioning interaction occurred in A and WUE. The interactions indicate that the sources responded differently as water stress progressed and that preconditioning influenced a source’s response to the imposed water stress. Significant (*p* = 0.01) between subject effects were detected for Ci and Ci/Ca among the seed sources.

The Iowa (black maple) and Ontario sources responded similarly in either preconditioning regime (Figure 2). The dry preconditioned Caddo, Missouri, and Tennessee sources as a group responded differently from moist preconditioned seedlings from these sources and from the Iowa and Ontario sources from either preconditioning regime, exhibiting a higher A, G_s_, and WUE together with a lower Ci/Ca during days 5–8 after withholding watering than the other source and preconditioning combinations (Figure 2). Dry preconditioned seedlings from the Iowa source initially declined more rapidly in A, G_s_, and WUE than moist-preconditioned seedlings of the Iowa source. However, after day 4, seedlings from the Iowa source under both preconditioning regimes declined similarly. The A, G_s_, and WUE of moist-preconditioned Caddo and Missouri seedlings declined more rapidly than dry preconditioned seedlings from those sources. The Caddo source typically maintained the highest A and G_s_ during the initial days of drying.

Significant differences (*p* < 0.001) were detected among seed sources with an ANOVA for all plant water relations parameters and gas exchange measurements on days 7 and 8 (Table 4 and Table 5). A significant (*p* < 0.001) source × preconditioning interaction also occurred in the A, G_s_, and E on days 7 and 8. The statistically significant interaction occurred because the Caddo and Missouri sugar maple sources responded differently to the two preconditioning regimes, in that the wet preconditioned seedlings from these sources had lower gas exchange than the dry preconditioned seedlings. In contrast, seedlings from the Ontario source and Iowa black maple source from either preconditioning regime responded similarly. WUE was greater in the dry preconditioned Caddo and Missouri sources. The Tennessee source had the highest A, G_s_, and, WUE, and curiously these maxima occurred in the moist preconditioned treatment.

#### 3.2.2. Diurnal Measurements

The A and G_s_ were highest in the dry preconditioned Caddo, Iowa, and Ontario sources and WUE was approximately similar for all sources from mid-morning to late afternoon during the initial few days after withholding watering (data not shown). As time increased, the A, G_s_, and WUE decreased more rapidly and the Ci/Ca increased more rapidly for the Iowa and Ontario sources than the dry preconditioned Caddo, Missouri, and Tennessee sugar maple sources. Diurnal response curves for G_s_ and E were similar to, and consistent with, A and WUE. The dry and moist preconditioned Ontario and Iowa sources responded similarly in measured gas exchange parameters. Moist preconditioned Caddo and Missouri seedlings declined more rapidly than dry preconditioned seedlings from these sources, but they responded similarly to the moist preconditioned Ontario and Iowa sources. Similar to the daily measurements, the Tennessee moist preconditioned seedlings maintained the highest diurnal A, G_s_, E, and WUE over the course of the imposed water stress through the last diurnal measurement on day 9.

### 3.3. Leaf Water Potential

Leaf water potential measurements did not differ between preconditioning regimes for RWC (*p* = 0.126), Ψ_100_ (*p* = 0.695), and Ψ_0_ (*p* = 0.505). So, the moist and dry preconditioned seedlings were pooled together for statistical analysis. The Ψ_100_ and Ψ_0_ were significantly different among the sources (Table 6). The Ψ_100_ was lower (*p* = 0.003) in the Iowa source (−1.51) and Ontario (−1.42) than in the Missouri (−1.21), Caddo (−1.18), and Tennessee (−0.98) sources. This pattern was similar with Ψ_0_ and lower (*p* = 0.003) in the Iowa source (−1.68) and Ontario (−1.56) than in the Missouri (−1.35), Caddo (−1.34), and Tennessee (−1.12) sources. The relative water content at zero turgor differed (*p* = 0.007) among sources being lowest in the Caddo sugar maple (93.1%) and highest in the Tennessee source (95.0%).

## 4. Discussion

This study revealed physiological and morphological patterns of response to water stress among sugar and black maple sources from locations with differing climatic conditions. Sugar maple ecotypes from warmer southwesterly locations (e.g., Oklahoma, Missouri, and Tennessee) responded similarly to imposed water stress. They were less sensitive to water stress than the more northerly Ontario and Iowa (black maple) sources. We hypothesized the Oklahoma source (Caddo) would exhibit the greatest resistance to water stress since the Caddo source is approximately 580 km southwest of the Missouri source and 770 km west of the Tennessee source. However, we did not find that to be the case, perhaps due to the relatively cooler and moister microclimate inside Red Rock Canyon where the Caddo sugar maple occurs as a relict sugar maple population dating from a cooler, moister period prior to 5000 y BP. The canyon microclimate is similar to the climate of forests containing sugar maple trees 300 km eastward near the Oklahoma and Arkansas border [47]. The long-term mean July temperature is similar among the southwestern sites (25 to 27 °C) and cooler in the more northerly study locations (21 to 22 °C). At least at the seedling level, there may be no difference among the Caddo sugar maple and other sugar maple trees from the species’ southwestern range. However, the Caddo sugar maple was shown to have superior water stress resistance to the “Legacy” sugar maple cultivar on dry sites in Kansas [48]. Even though the Caddo sugar maple failed to demonstrate superior water stress resistance over the other four sources, seedlings from this region, Missouri, and Tennessee did possess greater water stress resistance than sources from Ontario and Iowa (black maple).

### 4.1. Biomass Development and Allocation

Biomass portioning varied by seed source and preconditioning. Above ground biomass did not differ prior to water stress preconditioning. The black maple source grew more root mass, absolutely and proportionately, compared with seedlings of the other sugar maple sources. This finding was similar to that of Hilaire and Graves [19] with black maple from Iowa sources. We found that in response to mild water stress preconditioning, the black maple Iowa source responded by shifting biomass development to root systems proportionally more than did the other sources. The Iowa source had a greater R:S than the other sugar maple sources consistent with prior studies [19,23]. The Ontario and Iowa sources responded to mild water stress imposed through the dry preconditioning cycles by increasing their R:S, while the southwestern sugar maple sources did not. Graves [23] observed seedlings of black maple (from west central Iowa) to have a greater R:S than sugar maple from further north in Minnesota, both before and after imposed water stress. Sugar maple allocated greater carbon partitioning to leaf and stem tissue under moist conditions and black maple preferentially allocated to root growth. This was consistent with our findings. In contrast, the Caddo, Missouri, and Tennessee sugar maple sources responded alike with no significant change in their R:S or root mass when exposed to experimentally imposed water stress. Pallardy and Rhoads [49] also found no difference in the R:S of sugar maple seedlings from Missouri that were exposed to either continually moist soil or subjected to repeated drying cycles for ten weeks. The Ontario and Iowa seedlings were apparently more sensitive to water stress with respect to biomass allocation, having increased their R:S and lowered their SLA as a result of water stress preconditioning. It could also be surmised that the Caddo, Missouri, and Tennessee sources were not water stressed to the extent of the Ontario and Iowa sources due to greater efficiency in water use.

An increase in the R:S of woody plants often occurs in response to water stress [39]. Intrinsically high R:S or plastic increases due to water stress are mechanisms often associated with enhancing water stress resistance. However, the R:S is not always consistently higher in more water stress resistant tree species or ecotypes [39,50]. Water stress resistance is conferred by many different morphological and physiological traits [51]. In sugar maple, rooting patterns [39,52,53,54], leaf cuticle dimensions [2], leaf abscission [49], hydraulic lift [55], and mycorrhizal symbiosis are all factors that could influence water stress resistance of maples in nature but which were beyond the scope of this study.

The sugar and black maple sources had a similar SLA of approximately 200 cm^2^ g^−1^ (range 183 to 232 cm^2^ g^−1^) under moist preconditioning. Under dry preconditioning, the SLA decreased for the Ontario (171 cm^2^ g^−1^) and Iowa (144 cm^2^ g^−1^) sources. Plants with a lower SLA are characteristic of drought avoiders [51]. However, a leaf with a higher SLA coupled with a high photosynthetic rate provides a highly efficient assimilation system [56]. At the beginning of the water stress treatment, the Caddo sugar maple, which had a high SLA relative to other maples in this study, had a high rate of A. Thus, the Caddo sugar maple may have a highly efficient carbon assimilation system with respect to water use and this attribute might explain enhanced water stress resistance in the Caddo sugar maple.

### 4.2. Plant Gas Exchange

We showed that a northeasterly sugar maple source and black maple responded similarly. Our findings with the Ontario sugar maple and Iowa black maple source were similar to a finding by [19,57]. In that study, stomatal conductance decreased for black maple as a putative mechanism for drought avoidance. Our findings are not consistent with Ware [25,26] and Graves [23] who suggested that black maple trees from Iowa may have better water stress resistance. Our results and those of Pair [18] do not support the suggestion that black maple is a more water stress resistant maple. Pair [18] compared young sugar maple and black maple trees growing in Kansas over an 8-year period and found black maple seedlings and the black maple cultivar “Green Column” to have the least increase in stem caliper and height. In addition, during a drought Pair [18] found the black maple sources had the lowest pre-dawn and mid-day xylem water potential and also tended to have the most severe leaf scorch and intermediate leaf tatter. Nonetheless, increased root to shoot ratios, if they occur in sapling and mature black maple trees, could provide a mechanism to avoid drought, thus explaining multiple observations of this trait in mature specimens of black maple. 

We found G_s_ was maintained in the southwesterly sugar maple ecotypes more so than the Ontario and Iowa source. Hilaire [57] found water stress resulted in a 48% G_s_ decline in a black maple source from Iowa. Sugar maple stomata have been observed to close faster, open more slowly, and remain closed longer in response to water deficits in comparative studies [3,4,58,59,60,61]. Further, Lechowicz and Ives [62] found that sugar maple seedlings in a nursery that were subjected to mild water stress stabilized their internal CO_2_ concentration by varying their stomatal conductance, which suggests greater stomatal sensitivity to water stress. In our study, during the mid-portion of the imposed water stress the internal CO_2_ concentration increased faster in the Ontario and Iowa sources in comparison to the dry preconditioned Caddo, Missouri, and Tennessee sources.

The dry preconditioned Oklahoma and Missouri sources maintained a higher A and WUE during the mid-portion of the simulated water stress, declining less rapidly in comparison to their moist preconditioned seedlings. The Tennessee ecotype, regardless of preconditioning treatment, also declined less rapidly than other ecotypes. Preconditioning had no effect on these parameters for the Ontario or Iowa ecotypes. Ni and Pallardy [63] found the mesophyll in sugar maple from Missouri to be more sensitive to dehydration than post oak (*Quercus stellata*), because stomatal limitation decreased under water stress. The Ci of the sugar maple leaves increased and remained high even after 5 days of rehydration in their study suggesting mesophyll damage. Further, carboxylation efficiency remained low and the CO_2_ compensation point was higher after rewatering. It remains to be determined if ecotypic differences in mesophyll sensitivity to water stress for sugar and black maple exist.

### 4.3. Leaf Water Potential

Preconditioning can result in Ψ_0_ and Ψ_100_ changes [38,39,40]; however, preconditioning did not result in a difference between sources in Ψ_0_ and Ψ_100_ in this study, possibly since we imposed a mild water stress. We withheld watering until leaf wilting and then re-watered plants. It is possible that if we imposed greater water stress by letting seedlings persist in a wilted stage for several days, a preconditioning effect might have resulted in osmotic adjustment. As an adaptive feature, osmotic adjustment increases the ability to extract water from dry soil, increasing the ability to maintain cell turgor [39]. Osmotic adjustment in sugar maple has been observed by Kolb et al. [64] in a greenhouse seedling study (Pennsylvania source). In contrast, osmotic adjustment was not detected by Bahari et al. [5] in a forest field study (Missouri) nor by Tschaplinski et al. [65] with experimental greenhouse seedlings. Ellsworth and Reich [66] found the Ψ_0_ to differ among sugar maple trees growing in understory, gap, and clear-cut forest habitats, where trees in the sunnier clear-cut area had the lowest values. However, osmotic adjustment did not occur in sugar maple within a site during a drought the following year.

The similarity in leaf water potential with black maple from Iowa to an Ontario sugar maple source in this study is consistent with Pair [18] who found xylem water potential (pre-dawn and midday) were lowest in black maple sources and eastern sugar maple sources under water stress. These sources also had the greatest leaf tattering damage following a mid-summer drought [67]. Our results (−1.12 to −1.68) are within the range reported for Ψ_0_ of sugar maple trees grown under moist conditions (−0.81 to −2.18 MPa) and dry conditions (−0.97 to −2.44 MPa) [5,64,66]. The leaf development stage can influence Ψ_0_ with Tyree et al. [68] finding Ψ_0_ increased from −1.00 MPa in developing leaves to −2.00 MPa in mature leaves in sugar maple. The Iowa (black maple) and Ontario sources had the lowest Ψ_0_, suggesting they are less tolerant of water stress than the southwestern studied sources. All sources in this study were less negative than the mean −2.06 MPa (moist conditions) to −2.54 MPa (dry conditions) generally associated with North American tree species [40].

No significant difference in the relative water content at zero turgor (RWC_0_) occurred between moist or dry preconditioned seedlings and among sources. The sources had an average RWC_0_ of 0.944. This is near the 0.96 RWC_0_ observed by Kolb et al. [64] for greenhouse-grown sugar maple seedlings under moist conditions. In contrast, values for the RWC_0_ ranging between 0.77 and 0.90 have been reported for a field study of sugar maple in understory, gap, and clear-cut habitats [66].

The molecular research on sugar maple genetics has been mostly taxonomic, showing differences among related species and ecotypes [15,24]. While our study measured the morphological and physiological responses to imposed moisture stress preconditioning and moisture stress, there our molecular explanations behind the results [69]. Sugar maple has shown large-scale patterns with genetic variation and nuclear chloroplast data from western populations as detected in refugia populations as resulting from geologic events [69]. Yet, there appears to be little genetic difference, rather inducible responses to environmental conditions as imposed in this study between black and sugar maple [32]. St. Clair et al., [7] postulate a mechanistic model for moisture stress and the relationship to soil ion imbalances and nutrient uptake. Thus, a soil moisture limitation also leads to mineral stress in sugar maple, reduced carbon acquisition, and health decline in the species [6,70]. Future studies should look for genetic and molecular explanations for sugar maple tolerance to moisture stress, and maybe more importantly does this species have the molecular capacity to tolerate a potentially warmer and drier climate in the future.

## 5. Conclusions

Sugar maple ecotypes seedling from Oklahoma, Missouri, and Tennessee regulated water stress to a greater extent through gas exchange and greater water use efficiency in comparison to the Ontario and Iowa ecotypes. Our finding that black maple was more susceptible to water stress than southwestern sugar maple sources is consistent with the findings of Pair [18] and do not support a popular notion that black maple has generally greater water stress tolerance than other sugar maples, at least in the seedling stage or via leaf physiological mechanisms. However, if a high root to shoot ratio persists into maturity in black maple trees, the observations of greater water stress tolerance of this sugar maple subspecies might be explained. The agreement between our physiological assays of seedlings and the results of a study of water stress response of sugar maple cultivars in a field trial [18] indicate that seedling assays may be predictive of long-term physiological adaptations, affording sugar maple ecotypes drought and dry site tolerance. Finally, our results suggest that differences in WUE rather than inherent or induced differences in protoplasmic tolerance of water stress resulted in water stress hardiness differences among sugar maple ecotypes.

## Figures and Tables

**Table 1:** Sources of *Acer saccharum* subsp. *saccharum* and *Acer saccharum* subsp. *nigrum* (Iowa), approximate location, and climatic conditions.

Seed Source (Location)	Latitude (°N)	Longitude (°W)	Mean July Temp (°C)	Mean Annual Temp (°C)	Max Annual Temp (°C)	Min Annual Temp (°C)	Total Annual Precipitation (mm)
Oklahoma ^1^ (Caddo Co., OK, USA)	35.3736	−98.3775	27.4	14.9	22.1	7.9	849
Tennessee ^1^ (Haywood Co., TN, USA)	35.5894	−89.2586	27.0	15.8	21.4	10.2	1374
Missouri ^1^ (Texas Co., MO, USA)	37.5544	−91.8830	24.9	12.7	19.0	6.4	1141
Iowa ^1^ (Floyd Co., IA, USA)	43.0604	−92.6717	22.4	8.1	13.4	2.7	884
Ontario ^2^ (Ottawa, Canada)	45.3833	−75.7167	21.2	6.6	11.4	1.9	920

^1^ Climatological data (1981–2010) from National Oceanic and Atmospheric Administration accessed 15 March 2021 (https://www.ncdc.noaa.gov/cdo-web/) records or ^2^ Government of Canada Historical Climate Data accessed 15 March 2021 (https://climate.weather.gc.ca/) from a recording station in nearest proximity to seed source location.

**Table 2:** Morphological characteristics of 10-week-old *Acer saccharum* subsp. *saccharum* and *Acer saccharum* subsp. *nigrum* (Iowa) seedlings by source prior to preconditioning through moist or dry cycles. (*n* = 20).

Seed Source	Total Leaf Area (cm^2^) ^1^	Leaf Mass (g) ^1^	Stem Mass (g) ^1^	Shoot Mass (g)	Root Mass (g) ^1^	Root:Shoot Mass Ratio ^1^	Specific Leaf Area (cm^2^ g^−1^) ^1^
Caddo	319 (40.5)	1.21 (0.25)	0.26 (0.07)	1.47 (0.32)	0.27 (0.11) b	0.17 (0.03)	279 (24.7) b 212
Iowa	464 (112.8)	2.26 (0.59)	0.70 (0.33)	2.96 (0.89)	0.53 0(0.08) a	0.22 (0.04)	212 (15.1) a
Missouri	490 (60.7)	2.04 (0.31)	0.65 (0.12)	2.70 (0.42)	0.29 (0.04) b	0.11 (0.02)	243 (12.9) ab
Ontario	405 (83.0)	1.58 (0.30)	0.22 (0.02)	1.81 (0.31)	0.30 (0.05) b	0.17 (0.03)	253 (14.4) ab
Tennessee	363 (32.9)	1.26 (0.09)	0.47 (0.04)	1.78 (0.11)	0.22 (0.01) b	0.12 (0.01)	287 (6.7) b
Means	408 (32.1)	1.67 (0.17)	0.46 (0.08)	2.14 (0.24)	0.32 (0.04)	0.16 (0.01)	255 (8.8)
F(4,15) value	0.950	1.848	1.848	1.769	3.110	2.082	3.643
Model ^2^	0.462	0.172	0.172	0.188	**0.047**	0.134	**0.029**

^1^ Means (SE) in a column with same letter are not different (*p* > 0.05) with a Fisher’s Protected LSD. ^2^ Significance probabilities from the F statistic from an ANOVA. Significant values (*p* < 0.05) in bold.

**Table 3:** Morphological characteristics after 7 weeks of preconditioning for 17-week-old *Acer saccharum* subsp. *saccharum* and *Acer saccharum* subsp. *nigrum* (Iowa) seedling by preconditioning through moist or dry cycles. (*n* = 40).

Seed Source	Preconditioning Regime	Leaf Area (cm^2^) ^1^	Leaf Mass (g) ^1^	Stem Mass (g) ^1^	Shoot Mass (g) ^1^	Root Mass (g) ^1^	Root:Shoot Mass Ratio ^1^	Specific Leaf Area (cm^2^ g^−1^) ^1^	Height (cm) ^1^
Caddo	Dry	1216 ab (172)	5.40 abc (0.86)	2.14 a (0.65)	7.55 a (1.51)	1.33 ab (0.26)	0.19 ab (0.04)	228 ef (8.9)	57.6 (11.4)
Caddo	Moist	1415 ab (150)	6.82 abc (0.84)	3.15 ab (0.56)	9.97 ab (1.39)	2.69 bc (0.37)	0.27 b (0.02)	209 cde (8.3)	66.9 (5.9)
Iowa	Dry	1118 a (90)	7.83 cde (0.79)	3.33 ab (0.61)	11.16 ab (1.26)	4.69 d (0.83)	0.44 c (0.10)	144 a (4.4)	43.7 (8.1)
Iowa	Moist	1941 c (224)	10.53 e (1.51)	6.17 c (1.50)	16.70 c (2.95)	3.99 cd (0.89)	0.23 ab (0.02)	187 bcd (6.0)	91.6 (10.3)
Missouri	Dry	1440 abc (114)	6.97 abcd (0.48)	3.96 abc (0.30)	10.93 ab (0.35)	1.72 ab (0.20)	0.16 ab (0.02)	206 cde (7.8)	86.2 (4.8)
Missouri	Moist	1696 bc (238)	9.24 de (1.27)	5.14 bc (0.90)	14.39 bc (2.15)	2.61 bc (0.45)	0.18 bc (0.02)	183 bc (6.0)	81.2 (20.0)
Ontario	Dry	1280 ab (200)	7.39 bcd (0.82)	3.16 ab (0.41)	10.55 ab (1.16)	2.68 bc (0.55)	0.27 b (0.07)	171 ab (15.2)	55.8 (10.5)
Ontario	Moist	1544 abc (12)	7.40 bcde (0.52)	2.96 ab (0.00)	10.37 ab (0.53)	2.02 ab (0.33)	0.19 ab (0.02)	209 cde (13.2)	67.8 (16.0)
Tennessee	Dry	1096 a (172)	4.55 a (0.80)	2.53 a (0.61)	7.08 a (1.41)	0.89 a (0.19)	0.13 a (0.00)	244 f (8.5)	81.0 (7.8)
Tennessee	Moist	1078 a (188)	4.63 ab (0.69)	2.50 a (1.10)	7.13 a (1.79)	1.36 ab (0.36)	0.19 ab (0.01)	232 ef (13.7)	70.7 (20.5)
Means		1374 (67)	7.06 (0.40)	3.53 (0.30)	10.59 (0.68)	2.42 (0.24)	0.23 (0.02)	201 (5.6)	70.4 (4.2)
F (9,28) value		2.579	4.209	2.452	3.453	5.592	4.106	10.582	1.566
Model ^2^		**0.026**	**0.002**	**0.033**	**0.006**	**<0.001**	**0.002**	**<0.001**	0.174
Seed source ^2^		0.071	**<0.001**	**0.020**	**0.002**	**<0.001**	**0.002**	**<0.001**	0.358
Preconditioning ^2^		**0.014**	**0.044**	0.082	0.051	0.429	0.465	0.374	0.188
Seed source × Preconditioning ^2^		0.207	0.538	0.397	0.452	0.226	**0.015**	**0.002**	0.157

^1^ Means (SE) in a column with same letter are not different (*p* > 0.05) with a Fisher’s Protected LSD. ^2^ Significance probabilities from the F statistic from an ANOVA. Significant values (*p* < 0.05) in bold.

**Table 4:** Net photosynthesis (A, μmol CO_2_ m^−2^ s^−1^), leaf conductance to water vapor (G_S,_ mol H_2_O m^−2^ s^−1^), transpiration (E, mol H_2_O m^−2^ s^−1^), instantaneous water use efficiency (WUE, Ps × E^−1^), and intercellular to ambient CO_2_ concentrations (Ci/Ca, ppm ppm^−1^) on day 7 of withholding watering for *Acer saccharum* subsp. *saccharum* and *Acer saccharum* subsp. *nigrum* (Iowa) sources by preconditioning (Precond.) through moist or dry cycles. (*n* = 30).

Seed Source	Precond. Regime	A ^1^	G_S_^1^	E ^1^	WUE ^1^	Ci/Ca ^1^
Caddo	Dry	2.74 d (0.17)	0.039 d (0.001)	0.79 ab (0.02)	3.44 d (0.18)	0.65 a (0.02)
Caddo	Moist	1.73 c (0.04)	0.032 bc (0.001)	0.67 bcd (0.01)	2.60 c (0.10)	0.72 bc (0.01)
Iowa	Dry	0.86 a (0.16)	0.025 a (0.001)	0.52 d (0.02)	1.59 a (0.23)	0.82 ef (0.02)
Iowa	Moist	1.07 ab (0.20)	0.027 a (0.001)	0.57 cd (0.03)	1.80 ab (0.26)	0.80 def (0.02)
Missouri	Dry	2.56 d (0.33)	0.038 cd (0.004)	0.75 bc (0.06)	3.33 d (0.25)	0.67 ab (0.02)
Missouri	Moist	0.81 a (0.27)	0.025 a (0.002)	0.53 d (0.04)	1.27 a (0.40)	0.84 f (0.04)
Ontario	Dry	1.41 abc (0.13)	0.029 ab (0.002)	0.59 cd (0.04)	2.37 bc (0.10)	0.75 cd (0.01)
Ontario	Moist	1.50 bc (0.32)	0.030 ab (0.003)	0.58 cd (0.04)	2.42 bc (0.42)	0.76 cde (0.04)
Tennessee	Dry	2.51 d (0.28)	0.035 bc (0.003)	0.67 bcd (0.05)	3.67 d (0.24)	0.65 a (0.01)
Tennessee	Moist	3.87 e (0.07)	0.051 e (0.001)	1.00 a (0.01)	3.88 d (0.08)	0.62 a (0.01)
Means		1.91 (0.12)	0.033 (0.001)	0.67 (0.02)	2.64 (0.12)	0.73 (0.01)
F (9,80) value		20.148	13.487	17.040	13.103	12.531
Model ^2^		**<0.001**	**<0.001**	**<0.001**	**<0.001**	**<0.001**
Seed Source ^2^		**<0.001**	**<0.001**	**<0.001**	**<0.001**	**<0.001**
Preconditioning ^2^		0.112	0.845	0.869	**0.003**	**<0.001**
Seed Source × Preconditioning ^2^		**<0.001**	**<0.001**	**<0.001**	**<0.001**	**<0.001**

^1^ Means (SE) in a column with same letter are not different (*p* > 0.05) with a Fisher’s Protected LSD. ^2^ Significance probabilities from the F statistic from an ANOVA. Significant values (*p* < 0.05) bold.

**Table 5:** Net photosynthesis (A, μmol CO_2_ m^−2^ s^−1^), leaf conductance to water vapor (G_S,_ mol H_2_O m^−2^ s^−1^), transpiration (E, mol H_2_O m^−2^ s^−1^), instantaneous water use efficiency (WUE, Ps × E^−1^), and intercellular to ambient CO_2_ concentrations (Ci/Ca, ppm ppm^−1^) on day 8 of withholding watering for *Acer saccharum* subsp. *saccharum* and *Acer saccharum* subsp. *nigrum* (Iowa) sources by preconditioning (Precond.) through moist or dry cycles. (*n* = 30)

Seed Source	Precond. Regime	A ^1^	G_S_^1^	E ^1^	WUE ^1^	Ci/Ca ^1^
Caddo	Dry	2.01 b (0.23)	0.034 de (0.001)	0.71 cd (0.01)	2.81 c (0.29)	0.71 b (0.03)
Caddo	Moist	1.17 a (0.05)	0.027 bc (0.001)	0.60 bc (0.01)	1.95 b (0.09)	0.78 c (0.01)
Iowa	Dry	0.94 a (0.21)	0.025 abc (0.002)	0.55 ab (0.03)	1.58 ab (0.34)	0.82 cd (0.03)
Iowa	Moist	0.71 a (0.08)	0.024 abc (0.001)	0.52 ab (0.02)	1.36 ab (0.13)	0.84 cd (0.01)
Missouri	Dry	2.48 b (0.40)	0.036 e (0.004)	0.75 d (0.07)	3.12 c (0.31)	0.68 b (0.03)
Missouri	Moist	0.58 a (0.17)	0.022 ab (0.001)	0.52 ab (0.04)	0.99 a (0.27)	0.86 d (0.03)
Ontario	Dry	0.59 a (0.12)	0.021 a (0.001)	0.46 a (0.02)	1.23 ab (0.20)	0.85 d (0.02)
Ontario	Moist	0.94 a (0.24)	0.023 ab (0.002)	0.50 ab (0.03)	1.80 b (0.41)	0.80 cd (0.03)
Tennessee	Dry	1.86 b (0.42)	0.029 cd (0.004)	0.60 bc (0.08)	2.83 c (0.29)	0.71 bc (0.03)
Tennessee	Moist	3.87 c (0.15)	0.049 f (0.001)	0.99 e (0.02)	3.91 d (0.08)	0.61 a (0.01)
Means		1.51 (0.13)	0.029 (0.001)	0.62 (0.02)	2.16 (0.12)	0.77 (0.01)
F (9,80) ^2^		19.881	16.423	15.533	13.375	12.101
Model		**<0.001**	**<0.001**	**<0.001**	**<0.001**	**<0.001**
Seed Source		**<0.001**	**<0.001**	**<0.001**	**<0.001**	**<0.001**
Preconditioning		0.417	0.950	0.676	0.063	0.117
Seed Source × Preconditioning		**<0.001**	**<0.001**	**<0.001**	**<0.001**	**<0.001**

^1^ Means (SE) in a column with same letter are not different (*p* > 0.05) with a Fisher’s Protected LSD. ^2^ Significance probabilities from the F statistic from an ANOVA. Significant values (*p* < 0.05) bold.

**Table 6:** Relative water content at zero turgor (RWC_0_), osmotic potential at full turgor (Ψ_100_), and osmotic potential at zero turgor (_0_) (standard error of the mean).

Seed Source	Sample (n)	RWC_0_ (%) ^1^	Ψ_100_ (Mpa) ^1^	Ψ_0_ (Mpa) ^1^
Caddo	5	93.1 (0.5) a	−1.18 (0.09) bc	−1.34 (0.08) bc
Iowa	4	93.7 (0.5) ab	−1.51 (0.09) a	−1.68 (0.10) a
Missouri	5	94.9 (0.1) bc	−1.21 (0.09) bc	−1.35 (0.09) bc
Ontario	6	94.9 (0.3) bc	−1.42 (0.06) ab	−1.56 (0.06) ab
Tennessee	4	95.0 (0.6) c	−0.98 (0.08) c	−1.12 (0.09) c
Mean		94.3 (0.2)	−1.27 (0.05)	−1.42 (0.05)
F (4,19) ^2^		4.93	6.04	5.97
*p*-value		**0.007** ^2^	**0.003**	**0.003**

^1^ Means (*n* = 24) in the same column with a similar letter are not significantly different (*p* > 0.05) level using a Duncan test. ^2^ Significance probabilities from the F statistic from an ANOVA. Significant values (*p* < 0.05) in bold.

## Data Availability

Research data available through the corresponding author.
